# Supplementary material for: On the effect of flexible adjustment of the p value significance threshold on the reproducibility of randomized clinical trials
Source: PLoS One. 2025 Jun 13;20(6):e0325920. doi: 10.1371/journal.pone.0325920 (PMC12165351; doi:10.1371/journal.pone.0325920)
Supplement: S1 File — (PDF) [file pone.0325920.s001.pdf]

Supplementary Materials

Data Dictionary:

```
C               seriousness of type II to type I error, Eq. 1
O               prior odds that H1 is TRUE
pr              prior probability that H1 is TRUE, Eq. 2
d               the minimum effect size of interest
rep             number of repetitions in the Monte Carlo simulation
n, N            sample size in each arm
v               degree of freedom
s1              the standard deviation in the placebo group, assumed to be 1
s2              the standard deviation in the treatment group
err             number of errors when the conventional PST of 0.05 is used (Fig 1, orange column)
Err             number of errors when the conventional PST of 0.005 is used (Fig 1, red column)
ERR             number of errors when the flexible PST is used (Fig 1, green column)
ERR_            number of errors when the flexible PST is used assuming an equal variance
                (Fig 1, blue column)
not.converge    convergence flag for ERR
not.converge_   convergence flag for ERR_
```

Important parts of the R codes:

```
library("stats")
library("tidyr")
library("irr")
library("nleqslv")

#---------------------------------------------------
alpha <- function(t, v)
{
   return(2*pt(-abs(t), v, lower.tail=TRUE))
}
```

```
beta <- function(t, v, SNR)
{
#-- Power = PHI(-1.96-SNR) + 1 - PHI(1.96-SNR)
#-- beta = 1 - Power
   t = abs(t)
   return(pt(t - SNR, v, lower.tail=TRUE) - pt(-t - SNR, v, lower.tail=TRUE))
}

f_cost <- function(t, C, pr, v, SNR)
{
   return(C * pr * beta(t, v, SNR) + (1-pr) * alpha(t, v))
}

f <- function(t, C, pr, v, SNR)  #- first derivative of f_cost
{
   return(z(t, v) / (z(t+SNR, v) + z(t-SNR, v)) - C * pr / (2*(1 - pr)))
}

z <- function(x, v)              #- help to calculate the f
{
  return((1 + x^2/v)^(-(v+1)/2))
}


#-- Generate data, different s, pr = 0.5 -------------
set.seed(2024)
C = 1/4         #- seriousness of type II to type I error
pr = 0.5        #- the probability
O = pr/(1-pr)   #- corresponding odds to a probability of 0.5
d = 0.5         #- the minimum effect size of interest
rep = 10000     #- number of repetitions
N = c(50, 100, 200, 300, 500, 1000)
len = length(N)
```

```
dat = data.frame(pr = numeric(), d = numeric(), n = numeric(), s2 = numeric(),
                 err = numeric(), Err = numeric(), ERR = numeric(),
                 not.converge = numeric(), ERR_ = numeric(),
                 not.converge_ = numeric(), etype = character())

t_mat = t_opt = matrix(numeric(), nrow = 1, ncol=rep+2)

diff = 0    #-- Not different
for (n in N){
 err = Err = ERR = ERR_ = 0
 for(s2_ in c(0.5, 1.0, 2.0)){
   v = 2*n - 2
   se = sqrt(2/n)
   del = d / se
   opt = nleqslv(2, f, C = C, pr = pr, v = v, SNR = del)
   t_ = ifelse(opt$termcd == 1, opt$x, NA)  #- If converged?
   not.converge = not.converge_ = 0
   for (j in 1:rep){
     x1 = rnorm(n, mean = 0, sd = 1)
     x2 = rnorm(n, mean = diff, sd = s2_)
     W = t.test(x2, x1)                    #- Welch's t test
     t = as.numeric(W$statistic)
     v = as.numeric(W$parameter)
     se = (mean(x2) - mean(x1))/t

     err = err + ifelse(alpha(t, v) < 0.05, 1, 0)  #- Type I error
     Err = Err + ifelse(alpha(t, v) < 0.005, 1, 0) #- Type I error
     if (!is.na(t_)){
        ERR_ = ERR_ + ifelse(abs(t) > t_, 1, 0)    #- Type I error
     } else {                                      #- Not converged
        not.converge_ = not.converge_ + 1
     }
```

```
      s1 = sd(x1)
      del = d * s1 / se
      opt = nleqslv(2, f, C = C, pr = pr, v = v, SNR = del)
      t_opt[j] = ifelse(opt$termcd == 1, opt$x, NA)  #- If converged?
      if (!is.na(t_opt[j])){
         ERR = ERR + ifelse(abs(t) > t_opt[j], 1, 0) #- Type I error
      } else {                                       #- Not converged
         not.converge = not.converge + 1
      }
    }
    err = err/rep
    Err = Err/rep
    ERR = ERR/(rep - not.converge)
    ERR_ = ERR_/(rep - not.converge_)
    n.conv_ = not.converge_/rep
    n.conv = not.converge/rep
    dat = rbind(dat, data.frame(pr = pr, d = d, n = n, s2 = s2_, err = err, Err = Err,
                ERR = ERR, n.conv = n.conv, ERR_ = ERR_, n.conv_ = n.conv_, etype = "I"))
    t_mat = rbind(t_mat, c(s2_, n, t_opt))
  }
}

set.seed(2024)
diff = 0.5    #-- Different
for (n in N){
 err = Err = ERR = ERR_ = 0
 for(s2_ in c(0.5, 1.0, 2.0)){
    v = 2*n - 2
    se = sqrt(2/n)
    del = d / se
    opt = nleqslv(2, f, C = C, pr = pr, v = v, SNR = del)
    t_ = ifelse(opt$termcd == 1, opt$x, NA)
    not.converge = not.converge_ = 0
    for (j in 1:rep){
```

```
    x1 = rnorm(n, mean = 0, sd = 1)
    x2 = rnorm(n, mean = diff, sd = s2_)
    W = t.test(x2, x1)                      #- Welch's t test
    t = as.numeric(W$statistic)
    v = as.numeric(W$parameter)
    se = (mean(x2) - mean(x1))/t

    err = err + ifelse(alpha(t, v) >= 0.05, 1, 0)  #- Type II error
    Err = Err + ifelse(alpha(t, v) >= 0.005, 1, 0) #- Type II error
    if (!is.na(t_)){
       ERR_ = ERR_ + ifelse(abs(t) <= t_, 1, 0)    #- Type II error
    } else {
       not.converge_ = not.converge_ + 1
    }

    s1 = sd(x1)
    del <- d * s1 / se
    opt = nleqslv(2, f, C = C, pr = pr, v = v, SNR = del)
    t_opt[j] = ifelse(opt$termcd == 1, opt$x, NA)
    if (!is.na(t_opt[j])){
       ERR = ERR + ifelse(abs(t) <= t_opt[j], 1, 0) #- Type II error
    } else {
       not.converge = not.converge + 1
    }
  }
  err = err/rep
  Err = Err/rep
  ERR = ERR/(rep - not.converge)
  ERR_ = ERR_/(rep - not.converge_)
  n.conv_ = not.converge_/rep
  n.conv = not.converge/rep
  dat = rbind(dat, data.frame(pr = pr, d = d, n = n, s2 = s2_, err = err, Err = Err,
              ERR = ERR, n.conv = n.conv, ERR_ = ERR_, n.conv_ = n.conv_, etype = "II"))
  t_mat = rbind(t_mat, c(s2_, n, t_opt))
```

```
 }
}

t_mat <- t_mat[-1,]

t = data.frame(size = numeric(), s2 = numeric(), t = numeric())
for(i in 1:nrow(t_mat)){
  t = rbind(t, data.frame(size = rep(t_mat[i, 2], rep),
               s2 = rep(t_mat[i, 1], rep), t = t_mat[i, 3:ncol(t_mat)]))
}
t$logp = -log10(alpha(t$t, 2*t$size-2))

write.csv(dat, "RCT SS.csv", row.names = FALSE)
write.csv(t, "tp.csv", row.names = FALSE)
```
