# Supplementary material for: On the effect of flexible adjustment of the p value significance threshold on the reproducibility of randomized clinical trials
Source: PLoS One. 2025 Jun 13;20(6):e0325920. doi: 10.1371/journal.pone.0325920 (PMC12165351; doi:10.1371/journal.pone.0325920)
Supplement: Attachment — Submitted filename: Response to Reviewers R2.pdf [file pone.0325920.s004.pdf]

Dear Editor,

I would like to sincerely thank you for sharing the insightful and constructive comments from the second reviewer. I have carefully revised the manuscript in response to his valuable feedback. I believe the revised version of the manuscript has been significantly improved, and I hope you find it suitable for publication in *PLoS One*.

Thank you once again for your time and consideration.

Best Regards,
F. Habibzadeh, MD

Reviewer #2:

Thank you for your thoughtful and well-executed manuscript on the effect of flexible adjustment of the p-value significance threshold on the reproducibility of randomized clinical trials. Your approach offers a valuable contribution to the ongoing discussion surrounding statistical inference and reproducibility, particularly by proposing a flexible significance threshold based on minimizing a weighted sum of Type I and Type II errors.

While your manuscript is methodologically robust and generally well-presented, I would like to offer the following comments and suggestions to strengthen the manuscript further prior to publication:

Sensitivity Analysis of Prior Probability and Error Seriousness Coefficient

The current simulations assume a fixed prior probability (pr = 0.5) and a seriousness ratio (C = 0.25). While these values may reflect common scenarios in clinical research, their generalizability across fields is limited.

➤ I recommend adding a sensitivity analysis varying both pr (e.g., 0.2, 0.8) and C (e.g., 0.1, 1.0). This would enhance the robustness of your conclusions and demonstrate the flexibility and limitations of the proposed method under different decision-theoretic contexts.

Answer: Thanks for your great suggestion. I have added another Figure to show the results of different *pr* and *C* values.

Use of Pooled Variance under Heteroscedasticity

The simulations employ Student’s t-test with pooled variance, even in scenarios where variances between groups are unequal (s2/s1 ≠ 1).

➤ Please justify this methodological choice more clearly. Alternatively, consider including results using Welch's t-test as a robustness check. This would provide greater assurance that conclusions drawn under the assumption of pooled variance are not overly sensitive to violations of homoscedasticity.

Answer: Thank you very much for raising this important issue. I have changed the codes and used the Welch's *t* test. The Figures and results have been updated.

Convergence Validation of the Optimization Routine (optim)

Your method relies heavily on the optim() function to estimate flexible thresholds by minimizing a cost function. While this is an appropriate approach, the manuscript does not discuss whether convergence was reliably achieved or how non-convergence was handled.

➤ I suggest including either a brief description of the convergence diagnostics performed (e.g., convergence rate, error handling), or augmenting the code with a validation step to report non-converged iterations.

Answer: Thank you for raising this important issue. I changed the methodology, tried to solve the problem analytically, and finally changed the *R* codes. I checked the rate of convergence for each scenario. Fortunately, the optimization function used in the revised version (*nleqslv*) converged in all cases.
